# High-flow nasal cannula may be no safer than non-invasive positive pressure ventilation for COVID-19 patients

**DOI:** 10.1186/s13054-020-02892-9

**Published:** 2020-04-23

**Authors:** Kenneth E. Remy, John C. Lin, Philip A. Verhoef

**Affiliations:** 1 Pediatrics and Internal Medicine, Adult and Pediatric Critical Care Medicine, Washington University in St. Louis, St. Louis, MO USA; 2 Division of Pediatrics and Critical Care Medicine, Washington University School of Medicine, Saint Louis, MO USA; 3 University of Hawaii-Manoa, Kaiser Permanente Hawaii, Honolulu, HI USA

To the Editor:

We have read with great interest the *Surviving sepsis campaign: guidelines on the management of critically ill adults with coronavirus disease 2019 (COVID-19)* [1]. We are very concerned on the recommendation to use high-flow nasal cannula (HFNC) over non-invasive positive pressure ventilation (NIPPV).

We agree that HFNC has previously demonstrated reduced 90-day mortality compared to NIPPV in patients with acute hypoxemic respiratory failure [2] and that NIPPV has been demonstrated to have increased risk of aerosolized transmission to health care workers [3]. However, the differences in this risk with NIPPV compared to HFNC are largely unknown. Presently, it is known that COVID-19 (SARS-CoV-2) compared to SARS-CoV-1 remains viable in aerosols for at least 3 h with a marginal reduction in infectious titer from 103.5 to 102.7 TCID50 per liter of air [4]. Likewise, it showed a higher stability on plastic and stainless steel than on copper and cardboard, with virus viability seen up to 72 h on these surfaces [4]. This provides a concerning phenomenon for both HFNC and NIPPV as both interfaces are plastic with potential for aerosolization. An important difference is that the NIPPV interface provides a potential closed system (which may be advantageous) whereas HFNC allows patients to frequently touch their faces with continuous exposure to droplets, potentially increasing transmission to inanimate surfaces and hospital workers.

In 2019, Leung and colleagues found that HFNC use was not associated with increased air or contact surface bacterial contamination compared to simple oxygen mask in critically ill patients [5]. Unfortunately, viruses were not included in this study. Likewise, the term “aerosol” is a misnomer as it is well described that larger particle droplets can remain longer in circulation if ambient airflows (as in HFNC) sustain the infectious suspension for a longer duration. This, coupled with data from influenza infections showing aerosolized viruses are infectious at a lower dose than by nasal instillation, makes use of HFNC potentially worrisome [6]. The only known study evaluating SARS development in hospital workers was a retrospective study conducted prior to the widespread use of HFNC showing that development of SARS occurred in tracheal intubation (35%), HFNC 8%, and 38% (NIPPV) [3]; this suggests that both non-invasive (including HFNC) and invasive ventilation approaches carry significant risk.

Undeniably, HFNC provides more comfort to patients and likely improved compliance. However, since the data regarding transmission are unclear, we suggest, in addition to a negative pressure room, reverse isolation protection efforts with patients on HFNC wearing a mask over the nasal interface or a contained respiratory hood.

## Data Availability

Not applicable.
